# Unintended pregnancy and its associated factors among women with disabilities in central Sidama National Regional State, Ethiopia: a multilevel analysis

**DOI:** 10.1186/s12884-023-05848-3

**Published:** 2023-07-17

**Authors:** Zelalem Tenaw, Taye Gari, Achamyelesh Gebretsadik

**Affiliations:** 1 Department of Midwifery, College of Medicine and Health Sciences, Hawassa University, Hawassa, Ethiopia; 2 School of Public Health, College of Medicine and Health Sciences, Hawassa University, Hawassa, Ethiopia

**Keywords:** Disability, Unintended pregnancy, Prevalence, Associated risk factors, Ethiopia, Multilevel analysis

## Abstract

**Background:**

Unintended pregnancy is one of the most common reproductive health problems. The problem makes women with disabilities doubly burdened by their disabilities. The previous evidences are inconsistent and do not address all women with disabilities. The study aimed to assess the prevalence of unintended pregnancy and its associated risk factors among women with disabilities in Dale and Wonsho districts and Yirgalem city administration central Sidama National Regional State, Ethiopia.

**Methods:**

A community-based cross-sectional study design was conducted among 355 randomly selected women with disabilities living in the selected districts from June 20 to July 15, 2022. The data were collected through face-to-face interviews using a structured questionnaire. A multilevel logistic regression analysis model was employed to identify factors associated with an unintended pregnancy. The adjusted odds ratio (AOR) with a 95% confidence interval (CI) was used to report the measures of associations.

**Results:**

In this study, the prevalence of unintended pregnancy among women with disabilities was 65.6% (95% CI: 60.4, 70.6). After adjusting for potential confounding variables, middle economic status (AOR = 2.07; 95% CI: 1.02, 4.20), giving birth (AOR = 2.20; 95% CI: 1.21, 3.99), extremity paralysis types of disability (AOR = 0.26; 95% CI: 0.12, 0.57), living in urban residences (AOR = 0.22; 95% CI: 0.12, 0.40) and alcohol using (AOR = 0.28; 95% CI: 0.11, 0.74) were risk factors with unintended pregnancy.

**Conclusions:**

Unintended pregnancy among women with disabilities is remarkably high in central Sidama National Regional State, Ethiopia. Economic status, giving birth, types of disability, residence, and alcohol use were factors associated with an unintended pregnancy. As a result, economic empowerment, strengthening education and information about unintended pregnancy and its prevention strategies in rural settings are vital.

**Supplementary Information:**

The online version contains supplementary material available at 10.1186/s12884-023-05848-3.

## Background

Women with disabilities desire children in the same way that women without disabilities do, and they intend to have as many children as they can support [1]. Women with disabilities are discriminated against and excluded from various reproductive health services in the majority of developing countries, including Ethiopia [2, 3]. Due to different barriers, women with disabilities cannot access and use reproductive health services and could be exposed to reproductive health problems, mainly unintended pregnancy [4].

An unintended pregnancy is a pregnancy that occurs when a woman does not want to have a child at the time of conception [5]. Women with disabilities also suffer from unintended pregnancy and its effects, which makes them doubly burdened with their disabilities. They may go to unsafe abortion services to terminate the unintended pregnancy. Finally, they may lose their lives due to the complications of unsafe abortion [6].

A study conducted in the United States of America revealed that the proportion of unwanted pregnancies is higher (53%) among women with disabilities than among women without disabilities (36%) [7]. Another study from Cameroon also showed that unintended pregnancy among women with physical disabilities was common [8]. In our country, Ethiopia, the prevalence of unintended pregnancy among women with disabilities ranges from 15.4% [9] in Bahirdar to 67% in Addis Ababa [10, 11].

Different risk factors that are associated with unintended pregnancy among women with mental disabilities were identified by previous studies. A study conducted in South Africa marital status, level of education, ethnicity, and substance use as the main factors determining unintended pregnancy among women with disabilities [12].

The previous studies conducted in Ethiopia [9–11] did not identify the associated factors for unintended pregnancy among women with disabilities. There is also a disparity in the prevalence of unintended pregnancy. On the other hand, the previous studies had considered only urban women residents with disabilities and deaf and blind women with disabilities.

Therefore, this study aimed to determine the prevalence of unintended pregnancy and its associated risk factors among women with disabilities by considering rural and urban residency, all types of disability (except mental disability) and individual and community-level risk factors that are associated with an unintended pregnancy.

## Methods

### Study design and setting

A community-based cross-sectional study was conducted from June 20 to July 15, 2022. The study was conducted in Central Sidama Regional State, Ethiopia. The region report (2021) showed that the total population of the Central Sidama is 469,455 [13, 14]. Of these, 82,625 are people with disabilities. Based on the Labor and Social Affairs Office report (2021) and the WHO estimation [15], 19,207 people with disabilities are estimated to be of reproductive age. The details of the study settings were described in the previously published project [16].

### Population

Women with disabilities in central Sidama National Regional State were the source population. Women who have a pregnancy history or were currently pregnant and lived in the selected kebeles for at least six months were the study population, except those who cannot see and hear and are seriously ill during the data collection time.

### Sample size and sampling procedure

The sample size was determined by using Epi Info version 7 software with the following assumptions: a 95% confidence interval with a 15.4% prevalence of unintended pregnancy among reproductive-age women with disability [9] a level of significance (α) of 0.05, a 5% margin of error (d = 0.05), and a design effect of 1.64. The sample size for associated risk factors of unintended pregnancy was also computed using Epi-Info version 7 with the assumptions of a two-sided confidence level of 95%, a power of 80, a ratio of exposed to unexposed subjects, percent outcome in the unexposed group and percent outcome in the exposed group. Accordingly, the maximum (94) sample size was determined using substance use as a factor [12]. The sample size from the prevalence of 330 was larger than the associated factors’ maximum sample size of 94. After adjusting for an anticipated 10% nonresponse rate, the final sample size was 363. Then, the sample size was proportionally allocated to the 30 selected kebeles (20 rural and 10 urban) based on the number of women with disabilities who have a pregnancy history or are currently pregnant. Before conducting this study, a house-to-house census was conducted to determine the eligible women with disabilities in each kebele. The study participants were selected using a simple random sampling technique.

### Variables

The outcome variable was unintended pregnancy. Whereas the independent variables were marital status, educational status, ethnicity, and substance use (alcohol, chat) were the exposure variables.

### Data collection procedures and quality assurance

The data collection tool was adopted from the London Measure of Unplanned Pregnancy (LMUP) standard tool [17]. The data collection procedures and quality assurance were described elsewhere [16]. The trained data collectors did a pre-test on 19 (5%) women with disabilities who have a pregnancy history or are currently pregnant in Lokie and Tiltie kebele, Hawassa city, to check the tools, and corrections were made based on the feedback. The principal investigator (PI) monitored and controlled the overall process of data collection and made appropriate corrections for any issues that could be raised during data collection. The PI also checked the completeness of the questions daily.

### Outcome measurement

Unintended pregnancy was measured using the London Measure of Unplanned Pregnancy (LMUP) [17]. The tool had six questions (on contraceptive use, timing, intention, desire for a baby, partner discussion, and pre-conception preparations) through which women reported the circumstances of their current or recent pregnancy. Each item in the tool was scored 0, 1, or 2 according to the LMUP scoring guidelines [17]. The scores were summed across all six items, resulting in a total score of 0 to 12. Then the total LMUP scores of 0 to 3 are considered an unintended pregnancy [17, 18].

### Data management and analysis

Following collection, the data were imported into Stata version 16 for analysis using the “SSC install kobo2stata” command. The details of data management and analysis were described elsewhere [16]. The ICC of 0.16 and its chi-square (P < 0.001) significance level showed that using a multilevel analysis model is reasonable.

## Results

### Socio-demographic characteristics of study participants

A total of 355 women with disabilities participated in this study, with a 97.80% response rate. The ages of women with disabilities ranged from 15 to 48 years, with a mean (standard deviation) age of 31.25 (5.72) years. The majority (86.48%) of the study participants were married. 65% of women with disabilities had no formal education (were illiterate) and most (98%) of the study participants were not employed (Table 1).

**Table 1:** Socio-demographic characteristics of women with disabilities in Central Sidama Regional Stata, Ethiopia, 2022 (N = 355)

Variable		Number	Percent
Age in years mean (SD)		31.25(5.72)	
Religion	Protestant	277	78.03
	Orthodox	42	11.83
	Muslim	18	5.07
	Catholic	18	5.07
Marital status of the participants	Married	307	86.48
	Never married	48	13.52
Residency	Rural	214	60.28
	Urban	141	39.72
Participants educational status	Primary school	73	20.56
	Secondary school	42	11.83
	Vocational and technique	8	2.25
	Unable to read and write	232	65.35
Employment status	Employed	7	2
	Unemployed	343	98
Occupation	Have occupation	55	15.49
	No occupation	300	84.51
Self-perception	Good	261	73.52
	Bad	94	26.48
Wealth index of household	Low	124	34.93
	Middle	118	33.24
	High	113	31.83
Living with	Husband	281	79.15
	Family member	61	17.19
	Others^*^	13	3.66

_*_: Peers, relatives

### Prevalence of unintended pregnancy among women with disabilities

In this study, the prevalence of unintended pregnancy among women with disabilities was 65.6% (95% CI: 60.4, 70.6). Of these, 47.32% (95% CI: 42, 52.7) were women with hearing disabilities; 28.73% (95% CI: 24.08, 33.74) were women with vision disabilities; 20.56% (95% CI: 16.48, 25.15) were women with extremity disabilities; and 3.40% (95% CI: 1.76, 5.83) of women at wheel-chair.

### Factors associated with unintended pregnancy among women with disabilities

#### Random effect model

In the null model (model I), 16.11% of the variability in unintended pregnancy occurred at the community level (kebele level) and could be attributed to other unobserved community factors (ICC = 0.16), as supported by the chi-square (P < 0.001). This evidence also demonstrated the rationale for employing a multilevel analysis model.

### Fixed effect model

In the bivariable logistic regression, marital status, occupation, self-perception, economic status, giving birth, types of disability, alcohol use, chat use, and residence were associated risk factors for unintended pregnancy. But in the multivariable, multilevel logistic regression analysis, economic status, giving birth, types of disability, residence, and alcohol use were significantly associated with an unintended pregnancy.

Women with disabilities having a middle economic status had a twofold (AOR = 2.07; 95% CI: 1.02, 4.20) higher likelihood of unintended pregnancy than those with a low economic status. When comparing women who gave birth to those who did not the odds of an unintended pregnancy increased by twofold (AOR = 2.20; 95% CI: 1.21, 3.99) among those who gave birth. In terms of disability type, those with extremity disabilities had a 74% (AOR = 0.26; 95% CI: 0.12, 0.57) lower risk of unintended pregnancy compared with those with visual disabilities. Women with disabilities living in urban residences had 78% (AOR = 0.22; 95% CI: 0.12, 0.40) lower odds of unintended pregnancy compared with those living in rural residences. Those who use alcohol have a 72% (AOR = 0.28; 95% CI: 0.11, 0.74) lower likelihood of unintended pregnancy compared with women with disabilities who do not use alcohol (Table 2).

**Table 2:** Multilevel logistic regression analysis for factors associated with unintended pregnancy among women with disabilities in Central Sidama, 2022

Variables	Pregnancy			COR with 95% CI	AOR with 95% CI
	Wanted	Unintended			
Marital status	14	34	Never married	0.59 (0.27,1.29 )*	0.68 (0.31, 1.47)
	108	199	Married	1.00	1.00
Education	85	147	Illiterate	1.29 (0.77,2.18)	
	37	86	Literate	1.00	
Occupation	15	40	Yes	0.62 (0.31,1.26)*	0.79 (0.38, 1.66)
	107	199	No	1.00	1.00
Self-perception	38	56	Bad	1.55 (0.89,2.68)*	1.38 (0.73, 2.61)
	84	177	Good	1.00	1.00
Age (Years)	37	54	35 to 48	1.18 (0.46,3.03)	
	73	162	25 to 34	0.84 (0.34,2.05)	
	12	17	15 to 24	1.00	
Wealth index	52	61	Rich	2.06 (1.13,3.78)*	1.54 (0.82, 2.90)
	38	80	Middle	1.46(0.79,2.68)	2.07 (1.02, 4.20)**
	32	92	Poor	1.00	1.00
Gave birth	85	137	Yes	1.72(1.02,2.93)*	2.20 (1.21, 3.99)**
	37	96	No	1.00	1.00
Types of Disability	64	104	Hearing	0.87(0.44, 1.70)	0.67 (0.38, 1.18)
	13	60	Extremity	0.31(0.13, 0.78)*	0.26 (0.12, 0.57)**
	2	10	Wheel-chaired	0.39(0.07,2.09)	0.64 (0.11, 3.77)
	43	59	Vision	1.00	1.00
Alcohol use	7	55	Yes	0.24(0.10,0.57)*	0.28 (0.11, 0.74)**
	115	182	No	1.00	1.00
Chat use	6	26	Yes	0.43(0.16,1.17)*	0.97 (0.32, 2.97)
	116	207	No	1.00	1.00
Residence	24	117	Urban	0.24 (0.13,0.43)*	0.22 (0.12, 0.40)**
	98	116	Rural	1.00	1.00

*: P-value < 0.2; **: P-value < 0.05; AOR: Adjusted odds ratio; CI: Confidence interval;

## Discussion

The prevalence of unintended pregnancy among women with disabilities was 65.6%. After controlling for potential confounding variables, economic status, giving birth, disability types, residence, and alcohol use were found to be significantly associated with unintended pregnancy.

The prevalence of 65.6% in this study is almost similar to studies conducted in Addis Ababa, Ethiopia: 62.5% in 2011 [11] and 67% in 2017 [10]. However, it is much higher than a study conducted in Bahirdar City, Ethiopia (15.4%) [9]. The possible reason might be that the study in Bahirdar City did not use a standard unintended pregnancy measurement tool. It simply used one yes-or-no question to determine the magnitude of unintended pregnancy, which is prone to bias. The other possible reason might be the difference in data collection approaches. The Bahirdar City study used institution-based techniques, which may have missed the hidden majority of women with unwanted pregnancies in the community.

Regarding economic status, women with disabilities and middle economic status had a higher probability of having an unintended pregnancy compared with those with poor economic status. The possible justification might be that women with disabilities having a better economic income might increase their independence and freedom to enjoy sexual rights, which might expose them to unintended pregnancy. On the other hand, women with disabilities who gave birth had a higher chance of having an unintended pregnancy compared with those who did not give birth. The reason might be the fact that women who gave birth had an increased chance of sexual intercourse, which may have exposed them to unintended pregnancy [19] and could be due to unmet contraceptive needs [20]. Compared with women with vision disabilities, women with extremity disabilities had a lower probability of having an unintended pregnancy. Although it is difficult to compare a study from the United States of America due to socioeconomic and other differences, the national survey results revealed that unintended pregnancy was more common among women with vision disabilities than other disabilities [21]. The possible reason could be that women with extremity paralysis were considered asexual, physically unattractive, and not eligible for sexual intercourse. Due to this and the fear of sociocultural discrimination during pregnancy, the probability of having sexual intercourse and unintended pregnancy was lower than for women with vision disabilities [22, 23]. The other significant factor that is associated with an unintended pregnancy is the residential place of women with disabilities. Those who lived in the urban had a lower risk of having an unintended pregnancy compared with women with disabilities living in rural residence. The possible reason for the difference could be that, in rural residences, there is a high probability of a lack of access to information, transportation to the health facility, and contraceptive access compared with urban residences [24]. The other possible justification could be low socioeconomic status and the presence of sociocultural norms in the rural residence [24, 25]. Regarding alcohol use, women with disabilities who used alcohol had a lower chance of having an unintended pregnancy compared with those who did not use alcohol. This finding is in contrast with the studies conducted in South Africa [12, 26]. The possible reason might be that, in our study, the number of people exposed to alcohol was very limited (62 out of 355 people). Different evidence revealed that alcohol exposure might increase the probability of having sexual relations and unintended pregnancy [27, 28].

The findings of this study could be useful for governmental and non-governmental organizations to alleviate the burden of unintended pregnancy on women with disabilities, which makes them doubly burdened with their disabilities. This study attempted to address all women with disabilities who lived in either urban or rural settings, which may be used to demonstrate the magnitude and associated factors of unintended pregnancy among rural women with disabilities, who are frequently overlooked. The other important strengths of this study were the consideration of kebele level (level 2) factors associated with an unintended pregnancy and the use of a standard unintended pregnancy measurement tool known as the London Measure of Unplanned Pregnancy (LMUP) [17]. However, limitations should be taken into consideration while we are interpreting the results. The limitation of this study was the exclusion of women with mental disabilities and a lack of generalizability across all types of women with disabilities.

## Conclusions

Unintended pregnancy among women with disabilities is remarkably high in central Sidama National Regional State, Ethiopia. Economic status, giving birth, types of disability, residence, and alcohol use were factors associated with an unintended pregnancy. Therefore, informational communication and behavioural change are crucial to changing the risky behaviours of women with disabilities. It is also crucial to develop an appropriate strategy to address issues of unintended pregnancy and its prevention for women residing in rural settings.

## Electronic supplementary material

Below is the link to the electronic supplementary material.

Supplementary Material 1

## Data Availability

All data supporting this information is included in the main document.
