# Supplementary material for: Unintended pregnancy and its associated factors among women with disabilities in central Sidama National Regional State, Ethiopia: a multilevel analysis
Source: BMC Pregnancy Childbirth. 2023 Jul 17;23:522. doi: 10.1186/s12884-023-05848-3 (PMC10353093; doi:10.1186/s12884-023-05848-3)
Supplement: Supplementary file 1 — Supplementary Material 1 [file 12884_2023_5848_MOESM1_ESM.docx]

## Participant information

My name is Zelalem Tenaw Currently I am a PhD. Student at Hawassa University in public health and now I am researching reproductive health services (RHS) among people with disabilities (PWDs) in Dale and Wonsho districts, and Yirgalem city administration, Sidama National Regional State.

**Objective:** To provide new knowledge that could support the efforts to improve the RHSU of reproductive-age PWDs in SNRS, Ethiopia. You are selected randomly as a possible participant in this study.

**Potential risks:** There is no potential risk that may cause any harm to study participants.

**Benefits:** No financial benefits are related to this study. But by participating in this study, you contribute to improving reproductive health service utilization among PWDs.

**Confidentiality:** Your name will not be written in this form and will never be used in connection with any information you tell us. All information given by you will be kept strictly confidential. Your participation is voluntary and you are not obligated to answer any question which you do not wish to answer. If you feel discomfort responding to the questionnaire, please feel free to drop it. This questionnaire will take about 20 minutes.

Are you willing to participate in the study?

1- Yes 2 - No

If the answer is yes, thanks! Conduct the interview.

If the answer is no, Thanks! Don’t force or reinforce an individual to participate in the study

**Written consent form participants/family members/head of the household**

**Hawassa University**

I have read/heard and understood all about the objective and the process of the study. My participation is voluntary and not obligated to answer any question which I do not know or do not wish to answer. I also understood that all information given by me will be kept strictly confidential. Therefore I am willing to participate in this study.

Study participant sign _____________________________ date _____________

Data collector name: _________________________________________Signature _________

Date of interview ----------- Month --------------- /2014 E. C.

Supervisor’s Name ___________________________________________ Signature________

Checked date________________ /2014E.C.

Complete 1

Incomplete 2

Contact Address of the Principal investigator

Name: Zelalem Tenaw

E-Mail: [abigiatenaw@gmail.com](mailto:abigiatenaw@gmail.com)

Cell-Phone: +251-916415147

**Section I. Respondent’s socio-demographic characteristics**

This questionnaire is designed to collect information from respondents concerning reproductive health services and problems among PWDs in Sidama Regional State, Ethiopia.

Wereda: _________________ Kebele code: _________Participant code: ____________

| NO | Question | Response | | | Skip |
| --- | --- | --- | --- | --- | --- |
| 201 | Sex | Female | | |  |
| 202 | How old are you? | ____years ( in completed year) | | |  |
| 203 | What is your religion? | 1. Orthodox 2. Catholic 3. Protestant 4. Muslim 5. Other (Specify)___________ | | |  |
| 204 | What is your ethnic group | 1. Sidama 2. Amhara 3. Oromo 4. Wolayita 5. Guragie 6. Others (specify)_____________ | | |  |
| 205 | What is your marital status | 1. Never married 2. Married 3. Divorced/separated 4. Widowed | | |  |
| 206 | Residence | 1. Urban 2. Rural | | |  |
| 207 | Kebele | Mention: _____________________ | | |  |
| 208 | With whom you are living now? | 1. Husband/wife 2. Family member 3. Alone 4. Relatives 5. Friends/peers 6. Others (specify)__________________ | | |  |
| 209 | What is your educational status? | 1. Unable to read and write 2. Attended formal education | | |  |
| 210 | If attended formal education, specify the highest grade completed | ________Grade | | |  |
| 211 | What is your employment status? | 1. Employed 2. Not employed-------------------- | | | 213 |
| 212 | Types of employer | 1. Government 2. Non-governmental organizations (NGO) 3. Family members 4. Non-family member 5. Self-employed | | |  |
| 213 | Do you have an occupation? | 1. Yes 2. No ---------------------------------- | | | 215 |
| 214 | What is your occupation? | 1. Professional/technical/managerial 2. Clerical/religious 3. Sales and services 4. Skilled manual 5. Unskilled manual 6. Agriculture 7. Other (specify)______________ | | |  |
| 215 | What is your view about yourself? (Self-perception? | 1. Good self-perception 2. Bad self-perception 3. Not sure | | |  |
| 216 | Do you have community based health insurance? | 1. Yes 2. No ----------------------------------- | | | 301 |
| 217 | Mention types of community based health insurance | ______________________________ | | |  |
| **Section III: Participants and household wealth index:** Ask (and observe when possible) the following questions one at a time and write the response on the space provided or encircle the appropriate response from the options listed. | | | | |  |
| 301 | What is the main source of drinking water for members of your household? | Piped water:   - 1. Piped into dwelling   2. Piped to yard/plot   3. Public tab/standpipe   4. Borehole | | |  |
|  |  | Dug well:   1. Protected well 2. Unprotected well | | |  |
|  |  | Water from spring:   1. Protected spring 2. Unprotected spring | | |  |
|  |  | Surface water:   - - - 1. River/lake/pond/stream/dam | | |  |
| 302 | What is the main source of water used by your household for other purposes such as cooking and handwashing? | Piped water:   1. Piped into dwelling 2. Piped to yard/plot 3. Public tab/standpipe 4. Borehole | | |  |
|  |  | Dug well:   1. Protected well 2. Unprotected well | | |  |
|  |  | Water from spring:   1. Protected spring 2. Unprotected spring | | |  |
|  |  | Surface water:   1. River/lake/pond/stream/dam | | |  |
| 303 | Where is that water source located? | 1. In own dwelling  2. In own yard/plot  3. Elsewhere | | |  |
| 304 | How long does it take to go there, get water, and come back? | 1. Minutes __________ 2. Don’t know | | |  |
| 305 | In the past two weeks, was the water from this source not available for at least one full day? | 1. Yes 2. No 3. Don’t know | | |  |
| 306 | Do you do anything to the water to make it safer to drink? | 1. Yes 2. No ------------------------------------- | | | 308 |
| 307 | What do you usually do to make the water safer to  Drink?  Record all mentioned | 1. Boil 2. Add bleach/chlorine 3. Strain through a cloth 4. Use water filter (Sand/composite/etc.) 5. Solar disinfection 6. Let it stand and settle | | |  |
| 308 | What kind of toilet facility do members of your household usually use? | FLUSH OR POUR-FLUSH TOILET   1. Flush to a piped sewer system 2. Flush to septic tank 3. flush to a pit latrine 4. Flush to somewhere else 5. Flush, don't know where   PIT LATRINE   1. Ventilated improved pit latrine 2. Pit latrine with slab 3. Pit latrine without slab/open pit 4. composting toilet 5. bucket toilet 6. Hanging toilet/hanging latrine 7. No facility/bush/field | | |  |
| 309 | Do you share this toilet facility with other households? | 1. Yes 2. No | | |  |
| 310 | Including your household, how many households use this toilet facility? | 1. Less than 10 2. 10 or more 3. Don't know | | |  |
| 311 | Where is this toilet facility located? | 1. In own dwelling 2. In own yard/plot 3. Elsewhere | | |  |
| 312 | What type of fuel does your household mainly use for cooking? | 1. Electricity: 1. Yes, 2. No 2. Liquid petroleum gas: 1. Yes, 2. No 3. Natural gas: 1. Yes, 2. No 4. Bio-gas: 1. Yes, 2. No 5. Kerosene: 1. Yes, 2. No 6. Charcoal: 1. Yes, 2. No 7. Wood: 1. Yes, 2. No 8. Straw/grass: 1. Yes, 2. No 9. Agricultural crop: 1. Yes, 2. No 10. Animal dung: 1. Yes, 2. No 11. No food cooked in the house:1.Yes, 2.No | | |  |
| 313 | Is the cooking usually done in the house, in a separate building, or outdoors? | 1. In the house 2. In separate building 3. Outdoors 4. Other | | |  |
| 314 | Do you have a separate room which is used as a kitchen? | 1. Yes 2. No | | |  |
| 315 | Who is the owner of the house? | 1. Me 2. Rental 3. Family 4. Relative 5. Others (specify)_________________ | | |  |
| 316 | How many rooms in this household are used for sleeping? | --------Rooms | | |  |
| 317 | The main material of the roof of the house? | 1. Natural roofing (no roof, mud, and sod) 2. Rudimentary roofing (rustic mat/plastic shee, reed/bamboo, wood planks, and cardboard) 3. Finished roofing (metal/corrugated iron, wood, calamine/cement, ceramic tiles, roofing shingles) | | |  |
| 318 | The main material of the floor of the house? | 1. Natural floor (Earth/sand, dung) 2. Rudimentary floor (wood planks, and palm/bamboo) 3. Finished floor (parquet or polished wood, vinyl or asphalt strips/ plastic tiles, cement, ceramic tiles, carpet) | | |  |
| 319 | Does this household own any livestock, herds, other farm animals, or poultry? | Yes  No | | |  |
| 320 | How many of the following animals does this household own?  IF NONE, RECORD '00'.  IF 95 OR MORE, RECORD '95'.  IF UNKNOWN, RECORD '98'. | 1. Cows bulls_______________ 2. Other cattle______________ 3. Horses/Donkeys/Mules ____ 4. Camels________ 5. Goats _________ 6. Sheep _________ 7. Chickens/poultry ______ 8. Beehives ___________ | | |  |
| 321 | Do you have separate rooms for cattle? | 1. Yes 2. No | | |  |
| 322 | Does any member of this household own any agricultural land? | 1. Yes 2. No --------------------------------------- | | | 324 |
| 323 | How many hectares of agricultural land do members of this household own? | ___________hectares | | |  |
| 324 | Does any member of this household own: |  | Yes (1) | No (0) |  |
|  |  | 1. Electricity---------- 2. Radio--------------- 3. Television --------- 4. Non-mobile telephone---------- 5. Computer ---------- 6. Refrigerator ------- 7. Table---------------- 8. Chair --------------- 9. Bed with spring matters------------ 10. Electric mitad ----- 11. Kerosene lamp/pressure------ 12. Lamp--------------- |  |  |  |
| 325 | Does any member of this household own: |  | Yes  (1) | No  (0) |  |
|  |  | 1. Watch -------------- 2. Mobile phone------ 3. Bicycle------------- 4. Motorcycle/scooter- 5. Animal-drawn cart- 6. Car/truck ------------ 7. Boat with motor----- 8. Baggage ------------ |  |  |  |
| 326 | Does any member of this household have a bank account? | 1. Yes 2. No | | |  |
| 327 | Does any member of this household have a microfinance account? | 1. Yes 2. No | | |  |
| 328 | How often does anyone smoke inside your house?  Would you say daily, weekly, monthly, less often than once a month, or never? | 1. Daily 2. Weekly 3. Monthly 4. Less often than once a month 5. Never | | |  |

**Section III: Unwanted pregnancy questions:**  Please think of your current pregnancy (if currently pregnant) / your most recent pregnancy (if not currently pregnant) when answering these questions.

| NO | Question | Response | Skip |
| --- | --- | --- | --- |
| 701 | In the month that you became pregnant, were you and your husband used contraception? | 0. Always used contraception  1. Inconsistent use  2. Not using contraception |  |
| 702 | What do you feel about the timing of the pregnancy happened? | 0. Wrong time  1. OK but not quite right  2. Right time |  |
| 703 | Did you have an intention to become pregnant, Just before conception? | 0. Did not intend to become pregnant  1. Changing intentions  2. Intended to get pregnant |  |
| 704 | Have you wanted a baby, Just before conception? | 0. Did not want a baby  1. Mixed feelings about having a baby  2. Wanted a baby |  |
| 705 | Have you discussed with your partner about children, before conception? | 0. Had never discussed children  1. Discussed but no firm agreement  2. Discussed and agreed to pregnancy |  |
| 706 | Did you have health preparation (taking folic acid, stopping/reducing alcohol and smoking, seeking medical advice) before conception? | 0. No actions  1. Health preparations (1 action)  2. Health preparations (>2 actions) |  |
| 707 | Did you give birth? | - - - 1. Yes       2. No |  |
| 708 | How many live children do you have? | Mention :____________ |  |
| 709 | Did you use alcohol? | 1. Yes 2. No |  |
| 710 | Did you use chat? | 1. Yes 2. No |  |

**Thank you very much for your participation!!**
